# Clinical and economic impact of goal-directed fluid therapy during elective gastrointestinal surgery

**DOI:** 10.1186/s13741-018-0102-y

**Published:** 2018-10-04

**Authors:** Juying Jin, Su Min, Dan Liu, Ling Liu, Bixiao Lv

**Affiliations:** Department of Anesthesiology, the First Affiliated Hospital, Chongqing Medical University, 1 Youyi Road, Chongqing, 400016 China

**Keywords:** Goal-directed fluid therapy, Gastrointestinal surgery, Postoperative complications, Stroke volume

## Abstract

**Background:**

Several randomized controlled trials suggest that goal-directed fluid therapy (GDFT) may result in improved postoperative outcomes. The aim of this study was to assess the clinical and financial impact of the real-life implementation of intraoperative GDFT in patients undergoing elective gastrointestinal surgery in a Chinese tertiary medical center.

**Methods:**

This Quality Improvement Program (QIP) study comprised three phases of 5, 1, and 5 months, respectively. During the first phase, we retrospectively collected perioperative data from patients who received standard intraoperative fluid management from January to May 2016. Then a 1-month training period allowed the clinical staff to become familiar with the GDFT protocol. After the training phase, GDFT was used from July to November 2016. In the GDFT group, stroke volume (SV) was continuously monitored and optimized towards the plateau of the Frank-Starling curve. The primary outcome measure was postoperative morbidity (the proportion of patients with one or more complications within 30 days after surgery). Secondary outcomes were total hospital cost, postoperative length of hospital stay, and 30-day mortality.

**Results:**

Data from 200 patients before (control group) and 201 patients after the implementation of GDFT (GDFT group) were collected and compared. There was no significant difference in demographics and surgical procedures between the two groups. Postoperative morbidity was significantly lower in the GDFT group than in the control group (30.8% vs. 44.0%, *p* = 0.006). No significant differences were observed for mean total hospital cost (76,793 RMB vs. 74,444 RMB; *p* = 0.430), median postoperative length of hospital stay (10 days vs. 10 days; *p* = 0.104), and 30-day mortality (1% vs. 0.5%; *p* = 0.565).

**Conclusion:**

In patients undergoing gastrointestinal surgery, the implementation of a GDFT protocol was associated with a reduction in postoperative morbidity without increasing costs.

**Trial registration:**

clinicaltrials.gov, NCT02507557. Registered 13 July 2015.

## Background

Intraoperative fluid management is part of everyday anesthesiology practice and a key determinant of postoperative outcome. The advantages and limitations of liberal versus restrictive fluid therapy strategies have been debated for years (Brandstrup et al. 2003, Nisanevich et al. 2005, Futier et al. 2010). Insufficient fluid administration may be responsible for tissue hypoperfusion, organ dysfunction, and postoperative complications (Futier et al. 2010, Myles et al. 2018). Hypovolemia is not easy to detect from variables such as heart rate and blood pressure (Bennett-Guerrero et al. 1999, Thom et al. 2010). On the other hand, large volumes of intravenous fluid may cause complications due to tissue edema such as delayed healing and weaning from mechanical ventilation (Holte et al. 2002). As a result, goal-directed fluid therapy (GDFT) has been proposed to tailor fluid administration to the individual needs of patients undergoing major surgery (Bellamy 2006, Corcoran et al. 2012, Doherty and Buggy 2012, Calvo-Vecino et al. 2018).

Several randomized controlled trials (RCTs) and meta-analyses suggest that GDFT is useful to improve postoperative outcome, namely to decrease postoperative morbidity, hospital length of stay and costs (Pearse et al. 2005, Benes et al. 2010, Hamilton et al. 2011, Grocott et al. 2013, Scheeren et al. 2013, Zakhaleva et al. 2013, Calvo-Vecino et al. 2018). However, a limited number of RCTs have been done in China (Zhang et al. 2013, Zheng et al. 2013, Luo et al. 2017), and very few physicians and institutions have implemented this concept in their day-to-day practice. Importantly, the effectiveness of GDFT remains to be confirmed in real-life conditions. Therefore, we designed a Quality Improvement Program (QIP) to implement GDFT in patients undergoing major abdominal surgery and to study the impact on postoperative morbidity and costs.

## Methods

### Screening and consent

This study (clinicaltrials.gov Identifier: NCT02507557) was approved by the local ethics committee (registration number: 20160301) and was conducted in a tertiary teaching hospital. We studied consecutive adult patients who were to undergo elective gastrectomy, colorectal surgery, or small bowel resection, before (control group) and after (GDFT group) the implementation of GDFT. Patients less than 18 years old, without any comorbidity (American Society of Anesthesiologists physical status I), and pregnant women were excluded from this study. Patients were followed until 30 days after the operation or death. Written informed consent was obtained from all patients in the GDFT group. The local ethics committee waived the requirement for informed consent in the control group due to the retrospective nature of the analysis. All patients’ records/information were anonymized and de-identified prior to analysis.

### Study design

**Fig. 1:**
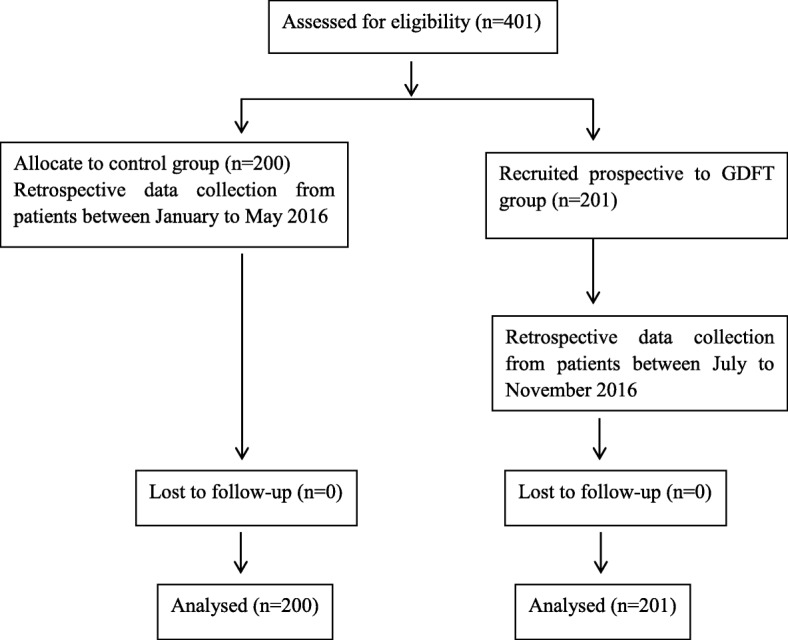
Flowchart of the patients in this study

Our QIP comprised three phases. During the first phase, from January to May 2016, patients received conventional intraoperative fluid management. In this control group, the use of fluids and vasoactive and inotropic drugs were at the discretion of the anesthesiologist. The second phase was conducted in June 2016. The goal of this training phase was to allow all members of our clinical staff to become familiar with the intraoperative GDFT protocol. After 1 month training, fluid management was conducted according to the GDFT protocol, from July to November 2016 (third phase, GDFT group; see Fig. 1).

### Anesthesia management

The patients in both groups were fasted for solid foods for 6 h and clear liquids for 2–3 h before surgery. All patients were given 300 mL oral drink (Outfast, Yichang Humanwell Pharmaceutical Co., Hubei, China) 2 to 3 h preoperatively. No preanesthetic medication was used. All patients received prophylactic antibiotics in accordance with established guidelines (Bratzler et al. 2013). Other principles of enhanced recovery after surgery (ERAS) protocol were not applied to patients undergoing gastrointestinal surgery within our institution during the execution of this study. In all cases, the anesthetic procedure was decided by the responsible anesthesiologist. General anesthesia was induced by 2–2.5 mg/kg propofol and 0.2–0.4 μg/kg sufentanil followed by 0.6–0.9 mg/kg rocuronium given for neuromuscular blockage. After tracheal intubation, patients’ lungs were ventilated (FiO_2_ 0.5–0.6) with a tidal volume of 8 mL/kg (ideal body weight) and an initial respiratory rate of 12 breaths/min adjusted to achieve an end-tidal CO_2_ between 35 and 45 mmHg. Anesthesia was maintained with inhalation of sevoflurane in 40% O_2_ in air. Intravenous sufentanil and rocuronium were injected intermittently as needed. Packed red blood cells were administered at the discretion of the anesthesiologist. Our perioperative care protocol suggests using a hemoglobin level of 7 g/dL as a transfusion threshold for healthy patients and 10 g/dL for patients with pulmonary or cardiac disease. Body temperature > 36 °C was maintained during surgery in both groups. Postoperative pain control was achieved with intravenous patient-controlled analgesia devices with tramadol.

### GDFT protocol

**Fig. 2:**
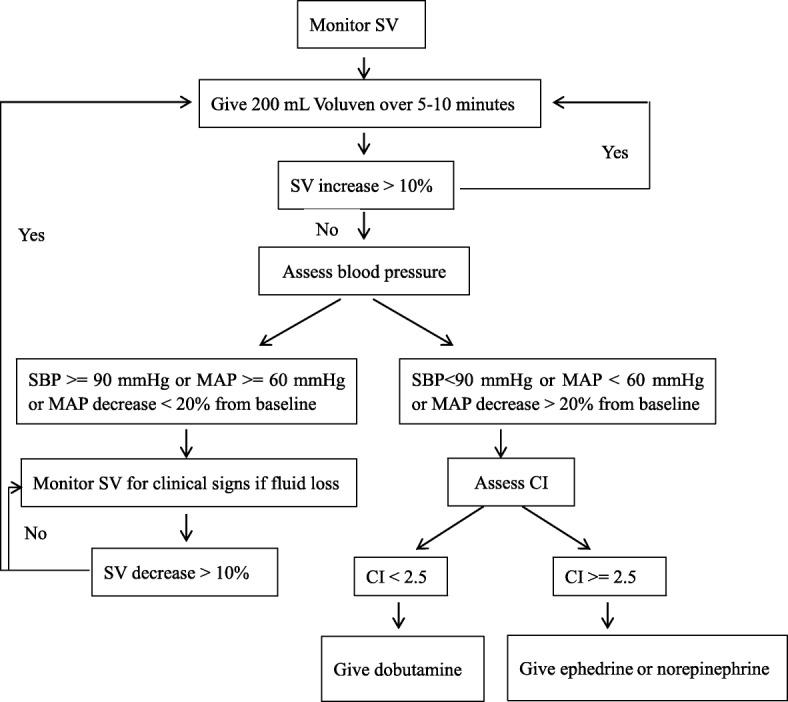
Intraoperative goal-directed algorithm. SV stroke volume, SBP systolic blood pressure, MAP mean artery pressure. Maintained dose of infusion of crystalloid during surgery was 2~ 4 mL/kg/h for open procedure and 1~ 2 mL/kg/h for laparoscopic procedure. After incision for open cases and after pneumoperitoneum for laparoscopic cases, patients received a 200-mL colloid bolus over 5–10 min. If SV increased by > 10%, the bolus was repeated until it increased by < 10%. Once reached, the SV plateau value was used as a target value during the entire surgical duration. Additional colloid boluses were given only if SV dropped by > 10% below the plateau value. In case of hypotension (SBP < 90 mmHg or MAP < 60 mmHg or MAP decrease > 20% from baseline) in fluid non-responders, an infusion of dobutamine was recommended if CI was < 2.5 L/min/m^2^, and ephedrine boluses of 5 to 15 mg or norepinephrine infusion were recommended if CI was > 2.5 L/min/m^2^. Protocol should not influence blood product administration

In the GDFT group, an arterial line was inserted into the radial artery of the non-dominant forearm before induction of anesthesia. The Vigileo/FloTrac system (Edwards Lifesciences, Irvine, CA, USA) was used to continuously monitor stroke volume (SV) and cardiac index (CI). Patients received 1 L of Ringer’s lactate solution right before anesthesia induction. Then, fluid maintenance with crystalloid was set at 2~ 4 mL/kg/h for open procedures (maximum dose of 400 mL/h) and 1~ 2 mL/kg/h for laparoscopic procedures (maximum dose of 200 mL/h). Boluses of IV colloid were given to optimise SV using the GDFT protocol recommended by the National Institute for Health and Care Excellence (NICE) in the United Kingdom (NICE 2011) (Fig. 2). After incision for open cases and after pneumoperitoneum for laparoscopic cases, patients received a 200-mL colloid bolus (Voluven, Fresenius Kabi, Bad Homburg, Germany) over 5–10 min. If SV increased by > 10%, the bolus was repeated until it increased by < 10%. Once reached, the SV plateau value was used as a target value during the entire surgical duration. Additional colloid boluses were given only if SV dropped by > 10% below the plateau value. In case of hypotension [systolic arterial pressure (SBP) < 90 mmHg or mean arterial pressure (MAP) < 60 mmHg or MAP decrease > 20% from baseline] in fluid non-responders, an infusion of dobutamine was recommended if CI was < 2.5 L/min/m^2^, and ephedrine boluses of 5 to 15 mg or norepinephrine infusion were recommended if CI was ≥ 2.5 L/min/m^2^. Adherence to the GDFT protocol was strongly encouraged but not systematically confirmed during the implementation phase.

### Outcome measures

The primary outcome measure was postoperative morbidity, defined as the percentage of patients with one or more complications within 30 days after surgery. Both postoperative in-hospital complications and complications that occurred after discharge and required ambulatory or in-hospital care up to day 30 after surgery were recorded. Postoperative complications included cardiac arrest (exclusive of death), myocardial infarction, stroke, pulmonary embolism, deep venous thrombosis, pneumonia, wound infection, wound dehiscence, urinary tract infection, ileus, anastomotic fistula, gastrointestinal bleeding, acute cardiac failure, acute respiratory failure, sepsis, unplanned reintubation, acute renal injury, hepatic dysfunction, and unplanned reoperation. Diagnosis and management of postoperative complications were undertaken by non-research staff according to our local practice. Secondary outcome measures included postoperative length of hospital stay, 30-day postoperative mortality, and total hospital costs. Costs are those associated with the actual procedures, as determined by the hospital using its accounting systems, and include both fixed and variable components. Both for the pre- and post-implementation periods, outcome data were extracted from our electronic medical records (EMR, Medicalsystem, Suzhou, Jiangsu, China). In order to guarantee that the data acquisition process was the same for the two study periods, data were collected retrospectively at the end of each period.

### Statistical analysis

Based on previous literature (Benes et al. 2010, Salzwedel et al. 2013, Zakhaleva et al. 2013), we estimated that complications may appear in 50% of our patients. Assuming a decrease in morbidity rate from 50 to 35%, a study sample size of 185 patients in each group was calculated for two-sided tests with type I error of 5% and power of 90%. Owing to an anticipated loss of about 10% of patients entering the study, we proposed to include 200 patients in each group.

Continuous normally distributed variables are expressed as mean ± standard deviation (SD), and non-normally distributed continuous variables are expressed as medians (interquartile ranges). Categorical variables are expressed as numbers and percentages. An independent sample *t*-test was used to test differences between groups for continuous normally distributed variables; a chi-square test was used for categorical data to test for differences between groups. When data were not normally distributed, a Mann-Whitney *U* test was used to analyze differences between groups. A level of *p* <  0.05 was defined as statistically significant. The statistical analysis was performed with the use of SPPS 17.0 for Windows (SPSS Inc., Chicago, IL, USA).

## Results

All eligible patients consented to participate in the study and thus 201 consecutive patients were prospectively recruited from July to November 2016 (GDFT group). They were compared with 200 patients who had surgery between January and May 2016 (control group). The relevant patient characteristics and surgical details are included in Table 1. There was no significant difference in age, gender, American Society of Anesthesiologists (ASA) physical status, surgical approach, surgical type, or duration of surgery between the two groups. A laparoscopic surgical technique was used in most of the cases.

**Table 1:** Patient demographics and surgical characteristics

	GDFT (*n* = 201)	Traditional (*n* = 200)	*p* value
Sex (male/female, *n*)	116/85	125/75	0.328
Age (mean ± SD, year)	62.7 ± 12.2	62.2 ± 12.3	0.697
Height (mean ± SD, cm)	161.1 ± 7.3	162.0 ± 7.5	0.243
Weight (mean ± SD, kg)	57.1 ± 9.9	60.3 ± 10.9	0.002
ASA physical status (II/III/IV, *n*)	140/58/3	154/44/2	0.248
Comorbidities			
Coronary artery disease (yes/no, *n*)	12/189	14/186	0.675
Hypertension (yes/no, *n*)	50/151	48/152	0.838
Diabetes mellitus (yes/no, *n*)	22/179	18/182	0.516
Surgical type (gastric/small bowel/colonic/rectal, *n*)	34//3/70/94	46/1/67/86	0.228
Surgical approach (open/laparoscopic, *n*)	23/178	21/179	0.763
Arterial line inserted (yes/no, *n*)	201/0	183/17	< 0.001
Surgical duration (median (IQR), min)	219 (180–268)	220 (180–260)	0.327

The volume of fluid administered is shown in Table 2. Patients in the GDFT group received on average 102 mL more crystalloid than the control group but the total volume of intraoperative fluid was comparable in both groups. Surgical blood loss was slightly but significantly higher in the GDFT group, who received more blood products than the control group. During surgery, urine output was higher in the GDFT group, resulting in a significant reduction in the intraoperative fluid balance. More patients received dobutamine in the GDFT group (Table 2).

**Table 2:** Intraoperative fluid administration and balance, use of vasoactive agents

	GDFT (*n* = 201)	Traditional (*n* = 200)	*p* value
Crystalloids (mean ± SD, mL)	1678 ± 361	1576 ± 466	0.014
Colloids (mean ± SD, mL)	672 ± 363	700 ± 289	0.390
Crystalloids + colloids (mean ± SD, mL)	2350 ± 572	2276 ± 612	0.210
Packed red blood cells (mean ± SD, mL)	48 ± 138	22 ± 90	0.022
Fresh frozen plasma (mean ± SD, mL)	13 ± 68	3 ± 24	0.047
Blood loss (mean ± SD, mL)	120 ± 162	90 ± 94	0.025
Urine output (mean ± SD, mL)	707 ± 466	467 ± 357	< 0.001
Net fluids balance (mean ± SD, mL)	1583 ± 562	1743 ± 571	0.005
Use of ephedrine (yes/no, *n*)	92/109	83/117	0.389
Use of norepinephrine (yes/no, *n*)	117/84	107/93	0.342
Use of dobutamine (yes/no, *n*)	44/157	14/186	< 0.001

Postoperative morbidity was significantly lower in the GDFT group (30.8% vs. 44.0%, *p* = 0.006). Total hospital cost, postoperative length of hospital stay, and mortality within the 30 days following surgery were comparable in both groups (Table 3).

**Table 3:** Postoperative morbidity and mortality, length of hospital stay, and total hospital costs

	GDFT (*n* = 201)	Traditional (*n* = 200)	*p* value
Cardiovascular complications	4 (2.0)	3 (1.5)	0.708
Cardiac arrest (exclusive of death, *n*, %)	0 (0)	0 (0)	–
Myocardial infarction (%)	0 (0)	1 (0.5)	0.315
Acute cardiac failure (%)	1 (0.5)	2 (1.0)	0.559
Stroke (%)	1 (0.5)	0 (0)	0.318
Pulmonary embolism (%)	1 (0.5)	0 (0)	0.318
Deep venous thrombosis (%)	1 (0.5)	0 (0)	0.318
Infectious complications	61 (30.3)	70 (35.0)	0.321
Pneumonia (%)	34 (16.9)	48 (24.0)	0.079
Wound infection (%)	23 (11.4)	17 (8.5)	0.325
Urinary tract infection (%)	3 (1.5)	4 (2.0)	0.698
Sepsis (%)	1 (0.5)	1 (0.5)	0.997
Gastrointestinal complications	12 (6.0)	15 (7.5)	0.541
Ileus (%)	6 (3.0)	3 (1.5)	0.315
Anastomotic fistula (%)	6 (3.0)	9 (4.5)	0.424
Gastrointestinal bleeding (%)	0 (0)	3 (1.5)	0.081
Respiratory complications	2 (1.0)	1 (0.5)	0.565
Acute respiratory failure (%)	1 (0.5)	1 (0.5)	0.997
Unplanned reintubation (%)	1 (0.5)	0 (0)	0.318
Other complications	11	17	0.234
Acute renal injury (%)	0 (0)	0 (0)	–
Hepatic dysfunction (%)	7 (3.5)	12 (6.0)	0.235
Wound dehiscence (%)	1 (0.5)	0 (0)	0.318
Unplanned reoperation (%)	3 (1.5)	5 (2.5)	0.471
Patients with one or more complications (%)	62 (30.8)	88 (44.0)	0.006
Readmission within postoperative 30 days (%)	5 (2.5)	9 (4.5)	0.430
Mortality within postoperative 30 days (%)	2 (1.0)	1 (0.5)	0.565
Postoperative length of hospital (median (IQR), day)	10 (8–14)	10 (8–13)	0.104
Total hospital cost (mean ± SD, RMB)	76,793 ± 26,522	74,444 ± 32,705	0.430
Total hospital cost (mean ± SD, $US*)	11,820 ± 4082	11,458 ± 5034	

*Exchange rate from June 22, 2018

## Discussion

This QIP study demonstrated that the implementation of GDFT was possible and effective in our institution. Indeed, it was associated with a significant reduction in postoperative morbidity and did not significantly increase costs.

Our findings are consistent with the results of several meta-analyses and randomized controlled trials which have demonstrated that GDFT is susceptible to improve postoperative outcomes in patients undergoing major abdominal procedures (Benes et al. 2010, Grocott et al. 2013, Zakhaleva et al. 2013, Zheng et al. 2013, Sun et al. 2017, Yuan et al. 2017, Calvo-Vecino et al. 2018). The beneficial effects of GDFT have been questioned in low-risk patients (Brandstrup et al. 2012) and during laparoscopic procedures (Senagore et al. 2009). Low-risk patients (ASA I) were excluded from our evaluation, but most of our patients had laparoscopic surgery, suggesting that GDFT may also have value in this context.

Before and after comparisons can provide valuable data regarding the effect of an intervention in real-life conditions, rather than under the stringent restraints of a randomized controlled trial (Saturni et al. 2014). A limited number of QIP studies have already investigated the effectiveness of GDFT implementation outside China (Cannesson et al. 2015, Habicher et al. 2016, Veelo et al. 2017). In Germany, Habicher et al. (Habicher et al. 2016) implemented GDFT in 130 patients undergoing hip revision arthroplasty and reported a significant decrease in postoperative morbidity. In the Netherlands, Veelo et al. (Veelo et al. 2017) compared the postoperative outcome of 100 patients undergoing esophagectomy before and after the implementation of a GDFT protocol. They observed a significant decrease in pneumonia, mediastinal abscesses, gastric tube necrosis, and ICU length of stay. In the USA, Cannesson et al. (2015) reported postoperative outcomes for 330 patients undergoing high-risk abdominal surgery and observed a decrease in postoperative morbidity from 39 to 25% after the implementation of GDFT. As of today, our study is therefore the largest QIP study investigating the effects of intraoperative GDFT on postoperative outcome. This is also the first one done in China, where surgical pathways and health care costs are different than in the USA and in Europe. Indeed, hospital costs reported in the manuscript are lower than those reported in the USA (Flynn et al. 2014, Michard et al. 2015) and in Switzerland (Vonlanthen et al. 2011).

Total intraoperative fluid volumes were not significantly different between the two groups, a finding consistent with the results of a meta-analysis of GDFT studies published in 2017 (Michard et al. 2017) and of the most recent multicenter GDFT randomized controlled trial (Calvo-Vecino et al. 2018). Our study design does not allow us to clarify why GDFT was more effective than our past practice. However, one may hypothesize that the individualization of fluid therapy is useful to ensure the right patients receive the right amount of fluid at the right time. Some patients likely received more fluid (because they were fluid responders) and others likely received less (because they were identified as non-responders) than they would have before the implementation of the GDFT protocol. This may explain why the average volume of fluid was comparable between groups (Michard et al. 2017). Differences in timing may also have played a role (Scheeren et al. 2013). However, because we did not study and record the timing of fluid boluses, we cannot draw any definitive conclusion regarding the influence of timing on postoperative outcome.

Interestingly, our study shows that implementing GDFT is a way to improve quality of surgical care without significantly increasing costs. Even if we did not specifically estimate the costs of complications, we can speculate that the cost for the hemodynamic equipment was offset by the reduction in postoperative morbidity and related costs. These findings are consistent with what has been reported by projection models (Bartha et al. 2012, Michard et al. 2015) and in prospective studies (Sadique et al. 2015), both in Europe and in the USA. This is an important message for hospitals that may be reluctant to invest in new technologies without knowing that it makes sense from a return on investment standpoint.

Our study has several limitations. First, given its “before and after” nature, we cannot claim causality between the GDFT intervention and the observed changes in postoperative outcome. Another potential disadvantage of such a design is the risk of imbalance between groups (Saturni et al. 2014). However, given the size of our study (> 400 patients), there was no significant difference at baseline between the GDFT and the control groups. A “before and after” study design also has advantages. It better represents daily clinical practice (Saturni et al. 2014). Indeed the control group is not influenced by the GDFT group. This is not the case in randomized controlled trials where the training and the Hawthorne effects inevitably affect the behavior of clinicians treating both groups (Habicher et al. 2016, Veelo et al. 2017). We believe that both randomized controlled trials and QIPs are useful and complementary, the former to demonstrate the value of new strategies, the later to confirm feasibility and efficacy in real-life conditions. Diagnosis of postoperative complications were undertaken by non-research staff according to our local practice, so that there was no official definition for each complication during the before implementation period. Proposing definitions for complications in the GDFT group only would have introduced a bias. Finally, if all patients from the GDFT group were equipped with a cardiac output monitor, we did not use—like in most GDFT studies—any tracking tools or target screens to optimize adherence to the hemodynamic protocol (Michard 2013). Therefore, we were unable to quantify the number of protocol violations, nor the time spent in target for hemodynamic variables, both factors that may have influenced our results.

## Conclusion

In summary, our study showed that real-life implementation of an intraoperative GDFT protocol was doable in our tertiary medical center and was associated with a significant reduction in the incidence of complications following gastrointestinal surgery. Interestingly, the observed improvement in quality of surgical care was not associated with a significant increase in hospital costs.

## Abbreviations

ASA: American Society of Anesthesiologists
CI: Cardiac index
EMR: Electronic medical records
GDFT: Goal-directed fluid therapy
MAP: Mean arterial pressure
NICE: National Institute for Health and Care Excellence
QIP: Quality Improvement Program
RCTs: Randomized controlled trials
SBP: Systolic blood pressure
SD: Standard deviation
SV: Stroke volume
